# Retraction Note: Association of the location and initial degree of malignant central airway stenosis with the risk of severe restenosis after interventional bronchoscopy

**DOI:** 10.1186/s12890-024-03147-x

**Published:** 2024-07-12

**Authors:** Saibin Wang, Renzhi Zhou, Siyao Zhu, Dan Yan

**Affiliations:** 1 Department of Respiratory Medicine, Jinhua Municipal Central Hospital, No. 365, East Renmin Road, Jinhua, 321000 Zhejiang Province China; 2 Shaoxing University School of Medicine, Shaoxing, 312000 Zhejiang Province China

**Retraction Note: BMC Pulmonary Medicine (2021) 21:323**

10.1186/s12890-021-01690-5

The Editor has retracted this article because, owing to an administration error in the editorial office, it was published after undergoing an initial editorial assessment, but before the peer review process had been undertaken. Post-publication peer review raised concerns that cannot be easily addressed by post-publication corrections as additional work is required. The authors have been invited to submit a new revised manuscript that will undergo robust peer review. The Publisher apologises to the authors for the administrative error.

